# *Leishmania spp* Epitopes in Humans Naturally Resistant to the Disease: Working Toward a Synthetic Vaccine

**DOI:** 10.3389/fcimb.2021.631019

**Published:** 2021-06-07

**Authors:** Magda Melissa Flórez, Rocío Rodríguez, José Antonio Cabrera, Sara M. Robledo, Gabriela Delgado

**Affiliations:** ^1^ Grupo de Investigación en Inmunotoxicología, Departamento de Farmacia, Facultad de Ciencias, Universidad Nacional de Colombia, Bogotá D.C., Colombia; ^2^ Secretaría Municipal de Salud, Municipio de Rovira, Tolima, Colombia; ^3^ Hospital San Vicente, Municipio de Rovira, Tolima, Colombia; ^4^ Programa de Estudio y Control de Enfermedades Tropicales (PECET)-Facultad de Medicina, Universidad de Antioquia, Medellín, Colombia

**Keywords:** synthetic peptides, reverse vaccinology, leishmaniasis, humans, naturally resistant, T epitopes

## Abstract

Vaccines are one of the most effective strategies to fight infectious diseases. Reverse vaccinology strategies provide tools to perform *in silico* screening and a rational selection of potential candidates on a large scale before reaching *in vitro* and *in vivo* evaluations. *Leishmania* infection in humans produces clinical symptoms in some individuals, while another part of the population is naturally resistant (asymptomatic course) to the disease, and therefore their immune response controls parasite replication. By the identification of epitopes directly in humans, especially in those resistant to the disease, the probabilities of designing an effective vaccine are higher. The aim of this work was the identification of *Leishmania* epitopes in resistant humans. To achieve that, 11 peptide sequences (from *Leishmania* antigenic proteins) were selected using epitope prediction tools, and then, peripheral blood mononuclear cells (PBMCs) were isolated from human volunteers who were previously divided into four clinical groups: susceptible, resistant, exposed and not exposed to the parasite. The induction of inflammatory cytokines and lymphoproliferation was assessed using monocyte-derived dendritic cells (moDCs) as antigen-presenting cells (APCs). The response was evaluated after exposing volunteers’ cells to each peptide. As a result, we learned that STI41 and STI46 peptides induced IL-8 and IL-12 in moDCs and lymphoproliferation and low levels of IL-10 in lymphocytes differentially in resistant volunteers, similar behavior to that observed in those individuals to *L. panamensis* lysate antigens. We conclude that, *in silico* analysis allowed for the identification of natural *Leishmania* epitopes in humans, and also STI41 and STI46 peptides could be epitopes that lead to a cellular immune response directed at parasite control.

## Introduction

Vaccines have decreased the incidence of several infectious diseases, especially those caused by bacteria and viruses because of either the generation of neutralizing antibodies or by the activation of memory T lymphocytes essential in pathogen elimination, and therefore allowing for the protection of the individual to reinfections (Sallusto et al., 2010).

One of the strategies available for vaccine development is reverse vaccinology that implies the use of pathogen and host OMICS to rationally design vaccine candidates. This approach has advanced significantly in vaccine research since the first full sequencing of a microorganism in 1995 (Fleischmann et al., 1995), up to the thousands of microbial genomes characterized so far. The above has allowed researchers to understand the polymorphisms between the pathogen species and the variability in human proteins involved in antigen presentation, therefore favoring a more accurate approach in the development of vaccines. These advances also allow bioinformatics tools to be more precise in their *in silico* screening performance, thus making research less time-consuming by reducing the possible candidates to be evaluated in *in vitro* and *in vivo* assays (Donati and Rappuoli, 2013; Grubaugh et al., 2013; Seyed et al., 2016).

T cell epitope prediction tools, based on human antigen presentation molecules, use algorithms that predict the binding of pathogen proteins to HLA molecules (Gfeller and Bassani-Sternberg, 2018).

Antigen searches made directly in humans increase the possibilities of success in candidate selection, although it is still a major challenge to reduce the gap between animal and human experimentation, as has happened in previous leishmaniasis vaccine attempts (Kedzierski, 2010). Depending on the endemic area and infecting specie, among other factors, between 20% and 91% of individuals exposed to the parasite rapidly resolve infection and the disease course becomes mild or even asymptomatic, these individuals are considered “naturally resistant” (evidenced by positive Leishmanin Skin Tests [LSTs], without any history of the disease) (Weigle et al., 1991; Das et al., 2014; Andrade-Narvaez et al., 2016; Best et al., 2018). Taking into account this information, T cell epitope screening in a naturally exposed population may open new possibilities to identify sequences linked to the natural resistance to the disease, which may be associated with the induction of protection toward the immunization of susceptible individuals.

Because *Leishmania* is an obligated intracellular parasite, T cell-mediated immune response plays an important role in the resolution of the disease. Although the immune response against the parasite has been described mainly in murine models, where there is a clear dichotomy between the protection mediated by Th1 response and susceptibility mediated by Th2 response (Belkaid et al., 1998; Belkaid et al., 2000), in humans such a dichotomy is not established. However, a predominant Th1 profile response, next to interleukin-12 (IL-12), interferon-gamma (IFN-ɣ), and tumor necrosis factor-alpha (TNF-α) production are essential to eliminate the parasite (Ajdary et al., 2000; Maspi et al., 2016). On the other hand, the IL-10 role is controversial because it has been identified as necessary to balance and avoid severe damage caused by an exacerbated immune response, but it has also been associated with the persistence of viable parasites after a clinical cure (Belkaid et al., 2002). The assessment of immune response in individuals “naturally resistant” to leishmaniasis would ease the comprehension of the immune response involved in resistance in humans, and therefore, it would guide the characterization of epitopes driving protection against the disease as potential vaccine candidates.

Therefore, in order to identify epitopes associated with leishmaniasis resistance: *(i)* we selected peptide sequences from *Leishmania* proteins, with a high probability to bind human HLA-DR haplotypes throughout *in silico* tools and *(ii)* we evaluated *ex vivo* antigenicity of those peptides in cells from individuals “naturally resistant” to the parasite, using monocyte-derived dendritic cells (moDCs) as antigen-presenting cells (APCs).

## Materials And Methods

### *In Silico* Analysis for T Epitopes

*Leishmania* spp. proteins were selected for *in silico* analysis considering: *i)* expression in amastigote parasite form, *ii)* high homology between *Leishmania* species causing cutaneous leishmaniasis in both old and new world, and *iii)* proteins previously reported as immunogenic *in vitro* and *in vivo*, with the induction of a Th1 response and/or reported as inductor of disease resistance, in different models. The selected proteins were: *i)* kinetoplastid membrane protein-11 (KMP-11, NCBI accession number AAF32344.1), *ii) Leishmania*-activated protein kinase C receptor (LACK, NCBI accession number AAG31685), *iii)* tryparedoxin peroxidase (TSA, NCBI accession number XP_010697482.1), *iv)* stress-induced protein 1 (STI1, NCBI accession number XP_010704221.1), and *v) Leishmania* eukaryotic initiation factor 4a (LEIF accession number XP_010696717.1). The T epitopes prediction tools were IEDB Analysis Resource (http://tools.iedb.org/mhcii/) (Wang et al., 2008; Wang et al., 2010), NetMHCIIpan (http://www.cbs.dtu.dk/services/NetMHCIIpan/) (Jensen et al., 2018), and Tepitopepan (http://datamining-iip.fudan.edu.cn/service/TEPITOPEpan/TEPITOPEpan.html) (Sturniolo et al., 1999; Nielsen et al., 2008; Zhang et al., 2009; Bordner and Mittelmann, 2010; Nielsen et al., 2010). Some 15-mer peptide sequences were selected by the following criteria: i) sequences with %Rank≥2 (strong binders) for at least two out of three prediction tools for more than one HLA-DR allele evaluated; ii) identical or similar sequences (synonym substitutions) between *L. panamensis*, *L. braziliensis*, and *L. major* species; and iii) sequences different from their human homolog when applicable. HLA-DR alleles taken in these predictions were based on studies of the most frequent alleles in the Colombian population (Trachtenberg et al., 1996; Correa et al., 2002; Reyes et al., 2007; Arias-Murillo et al., 2010; Ávila-Portillo et al., 2010; Yunis et al., 2013).

### Peptide Synthesis

Peptides were synthesized by the Chemistry Group belonging to Fundación Instituto de Inmunología de Colombia (FIDIC), based on Merriefield’s solid phase peptide synthesis methodology (Merrifield, 2006; Vanegas Murcia, 2014), using aminoacids T-BOC protected and purified by reverse-phase HPLC and mass spectrometry MALDI-TOF.

### Human Volunteers

Individuals included in this study were from different endemic areas in Colombia: *i)* rural area (La Luisa) of Rovira/Tolima, *ii)* PECET-UDEA research center, Medellín/Antioquia, and *iii)* Bogotá D.C. (non-endemic area, volunteers used as negative control). Human experiments were carried out following the Declaration of Helsinki. All volunteers included here agreed to participate through informed consent after the nature and possible consequences of the studies had been fully explained (approved by the ethical committee, act number 12, submitted on August 3, 2015, Science Faculty, National University of Colombia).

Humans were injected with 0.1 mL of LST solution (*Leishmania panamensis* preparation) by the intradermal route in the forearm, and the response was measured 72 h later. An induration diameter ≥4 mm was considered positive. Considering the clinical history of leishmaniasis and the LST result of each individual, they were classified into four clinical groups: *i*) susceptible to the disease (S n=10), including those with active cutaneous leishmaniasis and those cured after treatment, all of them with positive LST, all of them with a confirmed parasitological diagnosis; *ii*) resistant to the disease (R, n=10), characterized by a positive LST response without a history of leishmaniasis (no lesions history, nor leishmaniasis scars); *iii*) people living in endemic areas with a negative LST result (identified as individuals living in the same houses as susceptible volunteers, it means that they have been exposed to the same environmental risk factors), named exposed (E, n=14); and *vi*) not exposed individuals (NE, n=11), volunteers living in non-endemic areas with a negative LST response. It is important to highlight that the study place is not a T. cruzi circulation area, therefore it is a Chagas disease-free zone.

### Isolation and Differentiation of moDCs

Venous heparinized blood was collected, and PBMC´s (peripheral blood mononuclear cells) were isolated using density gradient reagent Lymphoprep™ (STEMCELL, Vancouver Canada). Cells were grown in RPMI 1640 medium (Invitrogen, USA), supplemented with inactivated fetal bovine serum (Hyclone, GE, Illinois, USA) 1% and incubated for 2 h at 37°C and 5% CO_2_. Afterward, the non-adherent fraction was recovered and preserved for posterior use, while the adherent fraction was stimulated with 1 ng/ml of IL-4rh (Invitrogen, USA) and 2 ng/ml of GM-CSFrh (Invitrogen, USA) every other day (2 pulses) for 5 days, to obtain moDCs.

### *In Vitro* Antigen Presentation

moDCs were pulsed with 10 µg/ml of each peptide or 10 µg/ml of *L. panamensis* lysate, or 25 ug/ml of PHA (Phytohaemagglutinin)–last two as controls–for 72 h; the supernatants were collected to assess inflammatory cytokines IL-8, IL-1β, IL-6, IL-10 TNFα, and IL-12p70 (CBA Becton Dickinson USA). After complete medium removal, moDCs were co-incubated for 7 days with the autologous non-adherent fraction (previously preserved) at a 1:10 ratio and stained with 5 µM of CFSE (Invitrogen, USA) in order to evaluate lymphoproliferation and the secretion of Th1-1h2-Treg cytokines IL-2, IL-4, IL-10, TNFα, and IFNɣ (CBA Becton Dickinson USA) in supernatants by flow cytometry (FACS Canto II BD).

### Data Analysis

Each result was normalized for each individual according to the Reactivity Index (RI), calculated by dividing the results of stimulated cells with each antigen between autologous cells without stimulation. The lymphoproliferation was evaluated as the Proliferation Index (PI) or Division Index (DI) calculated using FCS Express software. Data were analyzed by the non-parametric Kruskal-Wallis method to compare differences in cytokines and proliferation between all clinical groups. Graphs and statistical analyses were made using Graphpad Prism 7.0 software.

## Results

### *In Silico* Analysis

Under the *in silico* conditions described above, KMP-11, LACK, STI1, TSA, and LEIF proteins were pre-selected, and along the linear sequences, 1,483 overlapped 15-mer peptides were obtained overlapping 14 out of 15 amino acids in the next peptide. With each bioinformatic tool, a binding prediction for each peptide was run against 29 HLA-DR alleles (frequent in the Colombian population) resulting in 129,021 predictions, 43,007 for each tool. The results showed 73 sequences associated with high binding to any of the HLA-DR alleles evaluated. Finally, after considering the selection criteria previously described, 11 sequences met the established conditions (**Table 1**). None of the KMP-11 or TSA-derived sequences fulfilled the selection criteria, due to low binding prediction and high homology between parasite and human protein, respectively.

**Table 1:** *In silico* analysis of binding prediction to HLA-DR alleles.

Protein	Code	Sequence	Prediction tool			Identity between *Leishmania* species^§^	Homology (%) with human protein	Identity (%) with T. cruzi sequence**
			IEDB^‡^	NETMHCIIpan^‡^	TepitopePan^‡^			
**LACK**	LACK08	LEGHTGFVSCVSLAH	04:01, 04:04, 04:10, 11:01, 11:02, 13:05.	NB	04:01, 04:05, 04:07, 10:01.	100%	NO (33%)	60%
**STI1**	STI72	GRYVEAVNYFSKAIQ	11:02, 11:01, 13:01, 13:04.	NB	11:02, 13:04.	100%	NO (33%)	60%
	STI40	NWAKGYVRRGAALHG	01:02, 11:01, 13:04.	NB	08:02, 08:03.	100%	NO (53%)	80%
	STI41	KLSLLMLQPDYVKMV	01:02, 01:03, 03:01, 04:03, 04:04, 04:06, 04:11, 10:01, 15:01,	01:02, 04:03, 04:04, 04:06, 04:10, 04:11, 08:03, 12:01, 14:01, 15:01,	NB	100%	NO (20%)	73.3%
	STI42	ALTLMYLSGMKIPND	01:01, 01:02, 04:04, 11:01, 13:05, 15:01.	15:01.	01:02, 01:03.	100%	NO (27%)	46.6%
	STI43	TLYILNVSAVYFEQR	01:01, 01:02, 03:01, 04:10, 11:02, 12:01, 13:01, 13:02,	01:03, 04:07, 08:03, 13:02, 13:03, 14:02, 16:02.	NB	*93%	NO (53%)	53.3%
	STI44	YTIVAKLMTRHAFCL	08:03, 11:01, 11:02, 12:02, 13:03, 13:05, 14:01, 14:02, 16:02.	01:02, 04:04, 04:10, 04:11, 08:02, 11:01, 11:02, 12:01, 12:02, 13:01, 13:04, 13:05, 14:01.	NB	*80%	NO (33%)	73.3%
	STI46	SVKINKLISAGIIRF	01:03, 01:02, 03:01, 04:03, 04:04, 04:06, 04:11, 07:01, 08:02.10:01, 11:01, 11:02, 13:03, 14:01, 15:01.	01:01, 01:02, 01:03, 04:07, 07:01, 08:03, 11:02, 12:01, 13:01, 13:04, 14:01, 15:01.	NB	*93%	NO (40%)	80%
**LEIF**	LEIF73	FSIGLLQRLDFRHNL	13:05, 13:01, 11:01, 03:01, 13:04.	12:02.	12:02, 13:05.	100%	NO (47%)	93.3%
	LEIF50	YEIFRFLPKDIQVAL	01:02, 11:01, 11:02, 13:01, 13:04.	12:02.	13:03, 14:02.	100%	NO (47%)	86.6%
	LEIF52	QSVIFANTRRKVDWI	08:03, 11:01, 11:02, 13:03, 13:01, 14:01, 14:02,	11:01, 11:02, 13:01, 13:03, 13:04, 13:05.	NB	100%	NO (73%)	93.3%

‡Alleles with predicted strong binding to the sequence.

*Sequences with amino acids substitution for another with similar characteristics.

**^§^**L. panamensis, L. braziliensis, and L. major species.

NB, Non-binders.

**Trypanosoma cruzi strain CL Brener.

**Table 1** shows that, although the same cut off was applied for all of the tools, IEDB was identified as a strong binder of a higher number of sequences compared with NetMHCIIpan and TepitopePan, which, in a few cases, were identified as strong binders to the HLA-DRs evaluated. The results indicated that it is necessary to employ more than one *in silico* platform to improve precision for epitope identification. After *in silico* sequence selection, the 11 peptides were synthesized, and their biological evaluation was performed *ex vivo* in humans naturally exposed to infection by the *Leishmania* parasite. When we compared the peptide sequences with *Trypanosoma cruzi* proteins, we found a range between 46.6% and 93.3% of identity (**Table 1**), however considering that the samples were taken in a Chagas disease-free area, plus the absence of IgG against *T. cruzi* in the serum of all of the samples collected (data not shown), we can tell that the response observed in these individuals is linked to *Leishmania spp* exposure.

### Evaluation of Peptides Biological Response in Human Cells

#### Inflammatory Cytokine Production by moDCs

After the prediction of sequences (15-mer peptides) as probable T CD4^+^ epitopes, their antigenicity was evaluated in human cells isolated from volunteers belonging to the four clinical groups (**Table 2**).

**Table 2:** Classification of human volunteers.

Clinical group	Characteristics	n	Parasitological diagnosis	Leishmanin Skin Test (LST)	Gender*	Age in years old: mean (range)
**Susceptible (S)**	Affected by the disease	10	Confirmed	Positive	F:7M:3	44.1 (12-81)
**Resistant (R)**	No disease history	10	No lesions	Positive	F:6M:4	29.5 (18-55)
**Exposed (E)**	Living in endemic areas	14	No lesions	Negative	F:6M:8	47.6 (13-88)
**Not exposed (NE)**	Living in non-endemic areas	11	No lesions	Negative	F:7M:4	31.4 (23-57)
**Total**		45			F:26M:19	38.45 (12-88)

*F, female; M, male.

**Figure 1:**
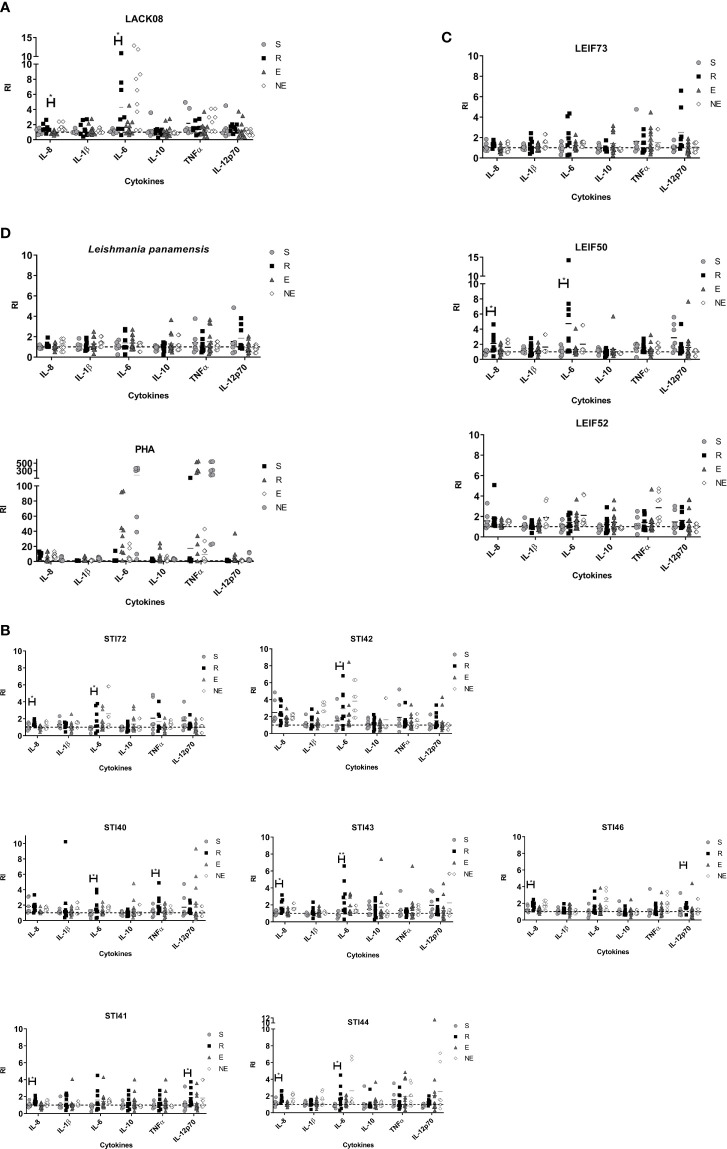
Cytokines detected in human moDCs supernatants from S (susceptible), R (resistant), E (exposed), and NE (not exposed) groups. Cell pulsed with **(A)** peptides derived from LACK protein; **(B)** peptides derived from STI1 protein; **(C)** peptides derived from LEIF, and **(D)** *L. panamensis* or PHA. P<0.05(*), P<0.01(**). Dashed line: non-stimulated cell response.

moDCs, obtained from each participant, were pulsed with either a single peptide or a control antigen, for 72 h, while cytokines IL-8, IL-1β, IL-6, IL-10, TNF-α, and IL-12p70 were measured in culture supernatant as an indirect measure of cell maturation. **Figure 1** shows the levels of cytokines produced by each clinical group in response to antigen stimulation. All antigens tested induced production of inflammatory cytokines by moDCs (**Figure 1**), without clear discrimination between the ones previously exposed to the parasite (LST+) and the ones who did not (LST-), however, the R group tended to produce higher IL-12 levels and to a lesser extend IL-6 when cells were stimulated with *L. panamensis* lysate (**Figure 1D**). When moDCs were exposed to each peptide, the R group secreted differential cytokines compared with the S group with the following stimulations: *i*) LACK08 induced higher levels of IL-6 (**Figure 1A**). *ii*) STI72, STI43 and STI44, LEIF50 induced higher secretion of IL-8 and IL-6; and *iii*) STI40 and STI42 promoted IL-6 and TNFα or IL-6 alone, respectively (**Figures 1B, C**).

The exposure of immature moDCs to the 15-mer peptides induces its apparent maturation, which is evident in its ability to secrete inflammatory cytokines into the culture supernatant in volunteers belonging to all clinal groups. It means that, as expected for the innate immune response, there was no preference depending on whether there was previous exposure to the pathogen or not. However, R group individuals produced significantly higher levels of proinflammatory cytokines against some peptides than those from the S group, behavior that could favor parasite control in the first ones. Although PHA has been reported as an inductor of proliferation and differentiation of lymphocytes (in this study we found that it modulates the immune response in immature moDCs) toward a predominant TNF-α response, followed by IL-6 and IL-8, and to a lesser extend IL-1β, IL-10, and IL-12, being mediated by PHA is a unique stimulus (**Figure 1D**).

#### Lymphoproliferation

After moDCs were stimulated with each antigen for 72 h, the cells were co-cultured with autologous lymphocytes for 7 days. PI represents the average number of cells that an initial cell became (ability to proliferate considering the overall population), and DI means the average number of cells that a dividing cell became (ability to proliferate of responding cells). Lymphocyte proliferation is generated as the result of specific recognition of its cognate antigen and preferentially observed in memory cells.

**Figure 2:**
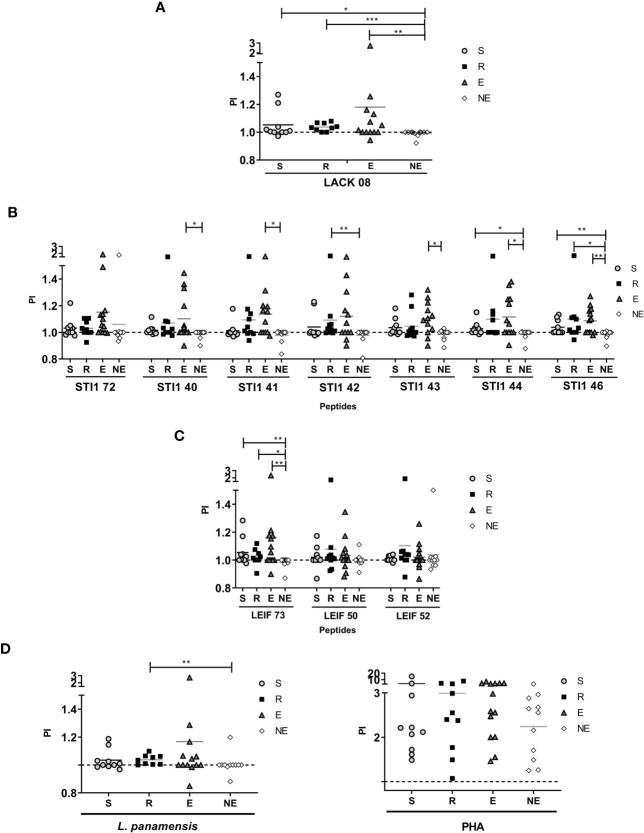
Proliferation Index (PI) evaluated in S (susceptible), R (resistant), E (exposed), and NE (not exposed) groups. Cells response to **(A)** peptides derived from LACK protein; **(B)** peptides derived from STI1 protein; **(C)** peptides derived from LEIF, and **(D)** *L. panamensis* or PHA. p<0.05(*), P<0.01(**), P<0.001(***). Dashed line: non-stimulated cell response.

**Figure 3:**
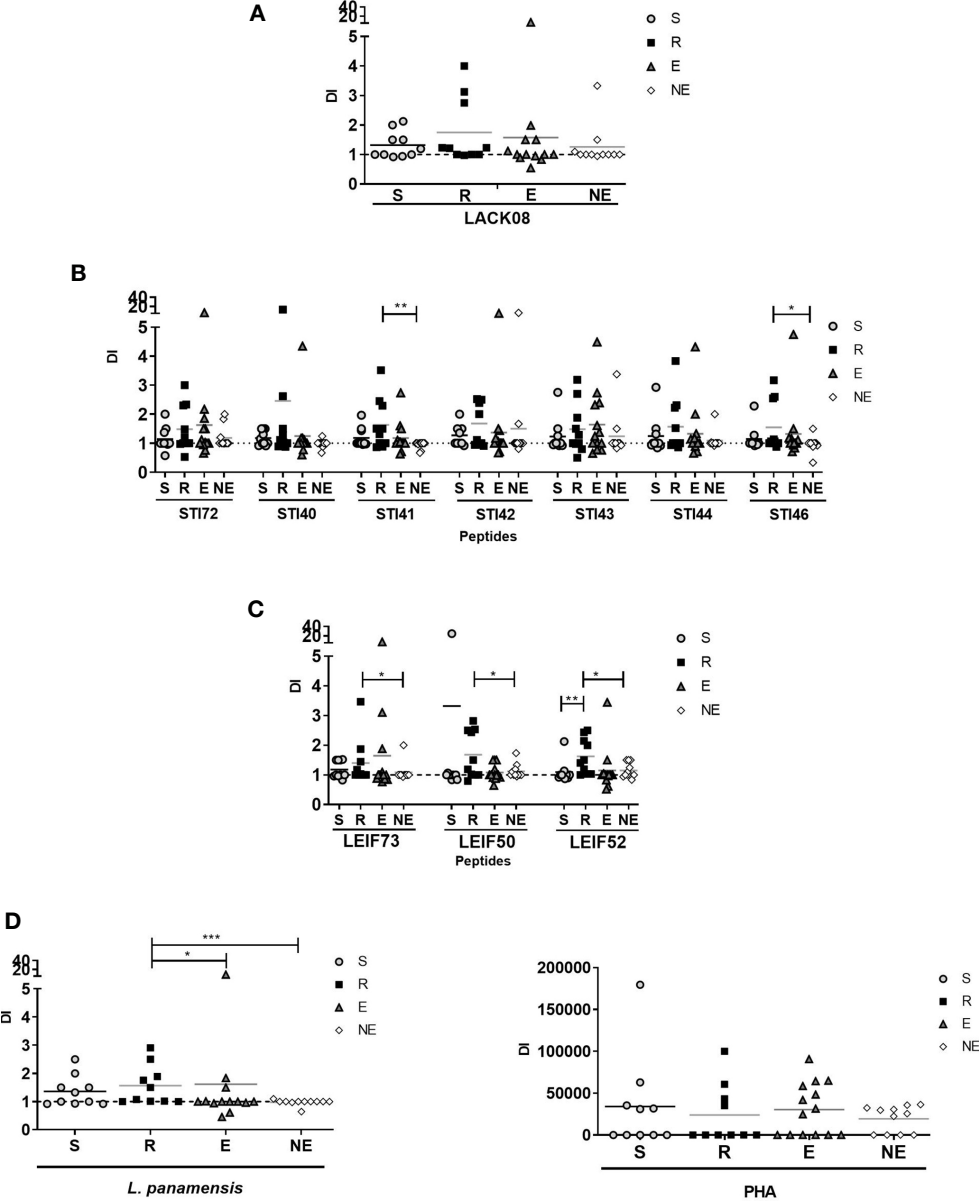
Division Index (DI) evaluated in S (susceptible), R (resistant), E (exposed), and NE (not exposed) groups. Cells response to **(A)** peptides derived from LACK protein; **(B)** peptides derived from STI1 protein; **(C)** peptides derived from LEIF, and **(D)** *L. panamensis* or PHA. p<0.05(*), P<0.01(**), P<0.001(***). Dashed line: non-stimulated cell response.

Proliferation observed with PHA stimulus showed that cells isolated from all the volunteers had the ability to respond (**Figure 2D**). Cells exposed to *L. panamensis* lysate showed that PI and DI were significantly higher in the R group than LST- participants. These results indicate that the difference between humans naturally resistant compared with those who present disease symptoms reflects a higher proliferative function of the lymphocytes that recognize parasite antigens (**Figures 2D** and **3D**). When proliferation was assessed with peptides stimulation, R and S groups responded in different levels of PI to each antigen, especially the R group which showed significantly higher PI with LACK08, STI42, STI46, and LEIF73 than the NE group (p<0,05); however, the E group also showed a predominant response against STI40, STI41, STI43, and STI44 (**Figures 2A–C**). These results could be due to an unspecific response, not restricted to a volunteer previously exposed to Leishmania (LST+), however, when DI was evaluated, the proliferation response was more specific in S and R groups, especially against STI41, STI46, LEIF73, LEIF50, and LEIF52, where DI was higher in the R group (behavior similar to *L. panamensis* lysate) (**Figures 3A–C**). Lymphoproliferation triggered by the APC-peptide complex indicates that these individuals have the capability to recognize that sequence as an epitope. The hypothesis is reinforced by the result in the NE group, which did not respond to most of the sequences derived from *Leishmania*. Also, some individuals from the E group showed proliferation (PI but not DI) against some antigens. Although LST in the E group was negative, such a response could be due to cross-reactions with phylogenetically related pathogens, circulating in the same geographical area, and therefore, the volunteers recognize homologous proteins. It is worth noting that there were no reports of Chagas disease in the zone where volunteers were included (PortalSivigila, 2019 Estadísticas de Vigilancia Rutinaria), so, cross-reaction with *Trypanosoma cruzi* is unlikely.

#### T Helper Profile Cytokine Production

**Figure 4:**
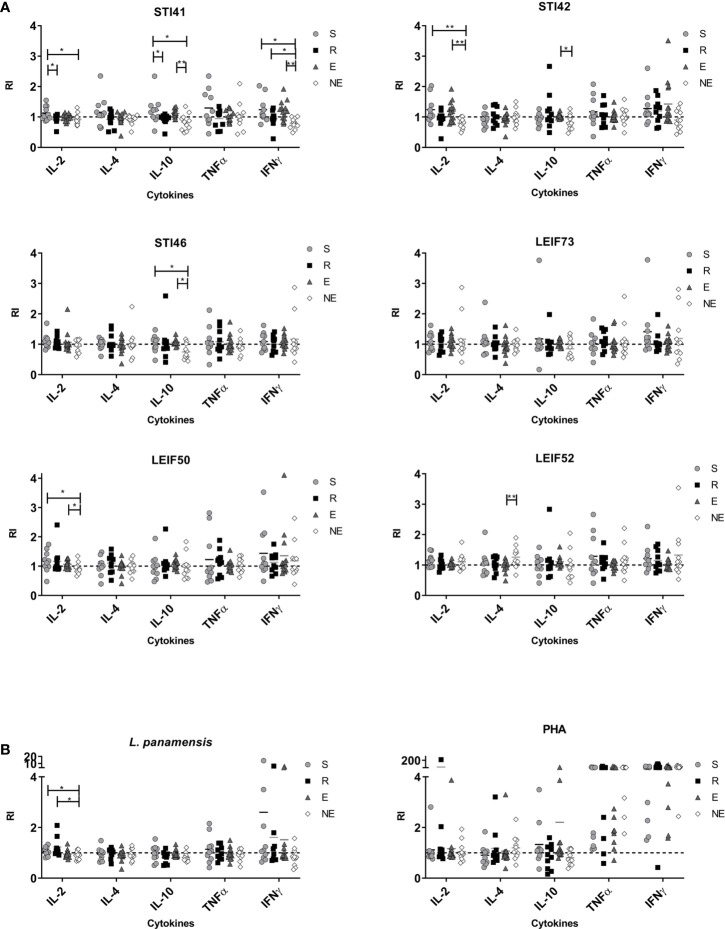
Cytokines from lymphocytes detected in culture supernatants in S (susceptible), R (resistant), E (exposed), and NE (not exposed) groups. Cell exposed to **(A)** Pre-selected peptides and **(B)** *L. panamensis* or PHA. p<0.05(*), P<0.01(**). Dashed line: non-stimulated cells response.

At the end of proliferation assay, supernatants were collected and Th1-Th2-Treg profile cytokines (IL-2, IL-4, IL-10, TNFα, and IFN-γ) were quantified. In general, PHA stimulation induced high levels of TNFα and IFN-γ, while peptide stimulation generated slight cytokine secretion (**Figure 4**). Considering that this work focused on the identification of epitopes related to disease resistance in humans, peptides that showed high PI or DI in the R group compared with NE were selected, therefore lymphocyte cytokines are described for STI41, STI42, STI46, LEIF73, LEIF50, and LEIF52 peptides (**Figure 4**).

Cells from group S individuals exposed to *L. panamensis* lysate produced small amounts of TNF-α and IFN-γ, while the R group produced IFN-γ and IL-2, predominantly. Focusing on the differences between the S group and R group, it is important to highlight that IL-2 was higher which correlated with proliferation in the latter group (**Figure 4B**). Additionally, there was a higher production (non-significantly) of IL-10 in the S group. With the previous information, we support the idea that the ability of an individual to produce IL-2 is related to a higher proliferative capability in lymphocytes, next to the secretion of Th-1 profile cytokines (especially IFN-γ) and low levels of IL-10, and could be generating a favorable activity against the parasite, promoting disease control.

Peptides STI41, STI42, and LEIF50, although they induced higher DI in the R group, IL-2 levels were lower than the S group (**Figure 4A**). STI41 and STI46 peptides did not produce detectable IL-10 levels in the R group, while the S and E groups did, showing similar behavior to cells exposed to *L. panamensis* lysate. Finally, cells exposed to LEIF73 and LEIF52 did not show important cytokine production or differences between clinical groups (**Figure 4A**).

Together all results obtained in this work, we could identify the STI46 peptide as a possible epitope associated with leishmaniasis resistance in human volunteers, because the R group mainly induced IL-8 and IL-12 at the time of antigen presentation, plus higher PI and lower IL-10 by lymphocytes than the S group. Cellular response activation associated with a pro-inflammatory profile that promotes intracellular parasite effective elimination such as *Leishmania* leads us to think that STI46 represents a prominent vaccine candidate that could be included in further *in vivo* studies and directed to evaluate its protective ability against leishmaniasis. The STI41 peptide induced IL-12 and IL-8 in moDCs from the R group, and IFN-γ and next to low IL-10 levels in lymphocytes. Although IL-2 is not detectable in supernatants—regardless of proliferation evidence—it could be evaluated in further assays to probe its usefulness as a vaccine candidate.

## Discussion

Reverse vaccinology strategies such as prediction by bioinformatic tools allow for the refining of a rational search for candidates to control infectious diseases. In order to increase probabilities to identify epitopes, it is necessary to apply more than one tool, each one with different algorithms—as we did in our study—thus favoring the discovery of natural epitopes in leishmaniasis or another infectious disease.

So far, leishmaniasis vaccines have been limited by a lack of correlation between animal models and humans, which is associated with the fact that most of the prototypes fail in clinical trial phases. For that reason, the identification of effective immune response directly in humans, and especially in those naturally resistant to the disease will provide more information about features that confer protection against leishmaniasis. In this study, LST+ individuals, although reflecting L*eishmania* cellular memory and *in vitro* lymphocytes proliferative capacity to whole parasite antigens, was lower in susceptible than naturally resistant individuals, pointing out that it could be one of the reasons why in some individuals, the disease does not progress.

Consequently, Division Indexes generated in lymphocytes are related to the number of generations that each stimulus prompted. The final clone count is the outcome of the TCR affinity, exposition time to the antigen-MHC complex, co-stimulation signals, as well as the surrounding cytokines and chemokines (van Stipdonk et al., 2008; Heinzel et al., 2018). Expansion potential against any antigenic stimulus depends on the lymphocyte profile itself. For example, Veiga-Fernandes et al. (2000) showed that, under the same circumstances, memory cells expand more and faster than naïve cells in an experimental murine model expressing the same TCR, at the same time a higher percentage of memory cells expressed effector cytokines and showed multifunctionality (Veiga-Fernandes et al., 2000). Also, Rogers et al. (2000) proved that low antigen doses caused a higher number of generations in memory T cells and showed higher cytokine secretion kinetics than naïve cells (Rogers et al., 2000), while Whitmire et al. (2008) reported that both memory and naïve cells start proliferation at the same time, but memory cells rapidly overcome its counterpart in numbers (Whitmire et al., 2008). Gudmundsdottir et al. (1999) demonstrated that IFN-ɣ production in a murine model is strongly related with cellular division (Gudmundsdottir et al., 1999), as well as Keshavarz Valian et al. (2013) who described in humans infected with leishmaniasis that cells that had more division cycles expressed higher IFN-ɣ levels compared with low division ones (Keshavarz Valian et al., 2013).

Considering that high PI and DI in response to some stimuli could reflect those epitopes which induced memory in resistant individuals—and therefore are considered *Leishmania* natural epitopes in humans who effectively control the disease—we propose that PI and DI could be used as vaccine candidate selection criteria and also as possible indicators of protection in experimental *in vivo* models. Additionally, peptides that induce Th1 cytokines next to IL-2 could also be considered as promising, because they could be related to cellular proliferative ability and its potential to generate memory cells.

Previous reports show that IL-10 induction next to the production of IFN-γ allows for parasite elimination and lesion resolution (Belkaid et al., 2002), however other reports, as well as in our study, suggest that high levels of IL-10 could be related to susceptibility to the disease (Stober et al., 2005; Rodrigues et al., 2014; De Luca and Macedo, 2016), as reported by Stober et al. (2005) in which after BALB/c mice were immunized with vaccine antigens, they identified a correlation between levels of IL-10 and the failure of the protection against parasite challenge with *L. major*. Additionally, by blocking IL-10 receptor with antibodies in vaccinated animals, those showed better resistance to the disease, suggesting that the IFN-γ/IL-10 relationship could be an indicator of vaccine success (Stober et al., 2005). Different from our results, Bittar et al. (2007) described lower proliferation in asymptomatic individuals and higher IL-10 production compared with those cured of the disease in cells exposed *in vitro* to *L. braziliensis* (Bittar et al., 2007). On the other hand, Trujillo et al. (2002) found similar lymphoproliferation responses and IFN-ɣ, IL-12, and IL-10 secretion between cured and asymptomatic individuals, after exposing PBMCs *in vitro* to *L. panamensis* (Trujillo et al. (2002), while Bosque et al. (2000) observed higher lymphoproliferation in asymptomatic compared with cured humans in PBMCs exposed to *L. panamensis* amastigotes (Bosque et al., 2000), similar to our work.

## Conclusion

*In silico* analysis, under established conditions, allowed for the identification of *Leishmania* natural epitopes in humans, a process that favors the wide screening of vaccine candidates for future studies and a reasonable selection of a lower number of those to be evaluated at the biological level. STI41 and STI46 peptides from Leishmania proteins were identified as promissory candidates to be included in further research and toward its analysis as potential synthetic vaccines against cutaneous leishmaniasis.

## Data Availability Statement

The raw data supporting the conclusions of this article will be made available by the authors, without undue reservation.

## Ethics Statement

The studies involving human participants were reviewed and approved by Approved by the ethical committee, act 12, august 3^th^ 2015, National University of Colombia, Science Faculty. The patients/participants provided their written informed consent to participate in this study.

## Author Contributions 

Project design: MF and GD. Development of laboratory methodology: MF. Approaching volunteers: RR, JC, and SR. Analysis and interpretation of data: MF and GD. Writing draft: MF. Review and editing: GD and SR. All authors contributed to the article and approved the submitted version.

## Funding

This work was funded by the “National project call for Research, Creation and Innovation strength”, Hermes code 37153 of Universidad Nacional de Colombia and also the principal author was financed by the Colombian Ministry of Science, Technology and Innovation, Colciencias-Colfuturo grant 6172-2014 for national Ph.D. students.

## Conflict of Interest

The authors declare that the research was conducted in the absence of any commercial or financial relationships that could be construed as a potential conflict of interest.
